# Transplant Trial Watch

**DOI:** 10.3389/ti.2022.10769

**Published:** 2022-09-01

**Authors:** John M. O’Callaghan

**Affiliations:** ^1^ University Hospitals Coventry and Warwickshire, Coventry, United Kingdom; ^2^ Centre for Evidence in Transplantation, University of Oxford, Oxford, United Kingdom

**Keywords:** systematic review, kidney transplantation, living donor liver transplantation, randomised controlled trial, synbiotics

To keep the transplantation community informed about recently published level 1 evidence in organ transplantation ESOT and the Centre for Evidence in Transplantation have developed the Transplant Trial Watch. The Transplant Trial Watch is a monthly overview of 10 new randomised controlled trials (RCTs) and systematic reviews. This page of Transplant International offers commentaries on methodological issues and clinical implications on two articles of particular interest from the CET Transplant Trial Watch monthly selection. For all high quality evidence in solid organ transplantation, visit the Transplant Library: www.transplantlibrary.com.

**Randomised Controlled Trial 1**

The Role of Renal Resistive Index as a Prognostic Tool in Kidney Transplantation: A Systematic Review.

*by Azzouz, S., et al. Nephrology Dialysis Transplantation 2022 [record in progress].*

## Aims

This study aimed to summarise the available evidence investigating the prognostic role of renal resistive index (RRI) in kidney transplant recipients (KTRs).

## Interventions

A literature search was performed on databases including MEDLINE, Cochrane CENTRAL, Embase and Scopus. Study screening and data extraction were conducted by two independent reviewers. The risk of bias was assessed using the Newcastle-Ottawa Scale for case-control studies and the Agency for Healthcare Research and Quality score for cross-sectional studies.

## Participants

26 studies were included in the review.

## Outcomes

Patient death, graft failure, measures of graft function and proteinuria.

## Follow-Up

Not applicable.

## CET Conclusion

This systematic review of Renal Resistive Index (RRI) in renal transplantation does have some good quality markers but also has some elements in the methodology and data quality that make strong conclusions difficult. The study was registered in advance with a review protocol on the PROSPERO system. A search was conducted on multiple database and 2 authors screened abstracts independently. Two authors extracted data and quality assessed studies independently. 26 studies were included, including 7049 renal transplant recipients, all studies were observational. 19 studies in languages other than English were excluded, as were an additional 19 studies for “erroneous data, unclear methods of analysis, or when data extraction could not be performed,” which is a very significant proportion of the data that could have been available. Meta-analysis was not possible due to significant heterogeneity in study design and outcomes within the remaining papers; Some studies reported RRI as a continuous variable and others as categorical, in others it is reported as median for a whole population. There was also inconsistency in the timing of RRI assessment after transplantation. Overall risk of bias was concluded to be moderate to high. Most studies that reported on death showed an association between higher RRI and risk of patient death, but this was not clearly associated with graft-related outcomes across the breadth of other studies. It may be that RRI is one representation of the patients’ overall health status rather than a graft-specific indicator. It is also possible that drawing firm conclusion from a disparate group of studies limits this review, particularly with the exclusion of a relatively large number of papers and data.

## Trial Registration

PROSPERO—CRD42020170822.

## Funding Source

No funding received.

**Randomised Controlled Trial 2**

A Randomized, Double-Blinded, Placebo-Controlled Trial Analyzing the Effect of Synbiotics on Infectious Complications Following Living Donor Liver Transplant—PREPRO Trial.

*by Mallick, S., et al. Journal of Hepato biliary pancreatic Sciences 2022 [record in progress].*

## Aims

Participants were randomly assigned to receive either the synbiotic drug Prowel^®^ or a placebo.

## Interventions

Participants were randomised into two groups: the intervention group, in which the patients participated in a personalised exercise rehabilitation program in addition to standard care, or the control group where the patients received standard care alone.

## Participants

100 recipients of live donor liver transplant (LDLT).

## Outcomes

The primary outcome was the occurrence of culture-proven bacterial infection in blood, urine or drain fluid. The secondary outcomes included hospital stay, noninfectious complications, use of antibiotics and 30-day mortality.

## Follow-Up

30 days.

## CET Conclusion

The double-blinded, randomised controlled trial evaluated if 2-weeks of synbiotic therapy starting 2 days before living donor liver transplantation (LDLT) reduced infections in recipients. LDLT recipients were randomised according to a computer-generated sequence in sequentially numbered envelopes to the synbiotic drug or an identical looking placebo. The power calculation showed that 100 patients were needed. One hundred patients were randomised and all were included in the 30-day posttransplant analysis of primary and secondary outcomes. There were significantly less infections in the synbiotic group compared with placebo. Further analysis showed that blood stream infections were lower in the synbiotic group but there were no differences between groups for urinary tract and intra-abdominal infections. All secondary outcomes were similar between groups.

## Jadad Score

5.

## Data Analysis

Strict intention-to-treat analysis.

## Allocation Concealment

Yes.

## Trial Registration

CTRI/2017/09/009869.

## Funding Source

No funding was received.

## Clinical Impact Summary

This is overall a well conducted randomised controlled trial in live donor liver transplantation. There are some slight weaknesses in the methodology on deeper assessment; The method of randomisation was computer-generated however the results were kept in sealed envelopes, so this is not completely free of bias potential. The study is described as double-blinded, with capsules used to convey the study symbiotic preparation, or emptied capsules for the placebo arm of the study. However, it is possible that patients or clinicians could then determine which arm of the study they were in by closely examining the capsules. Reassuringly the primary endpoint was well-defined, as the presence of culture-proven bacterial infection in the blood, urine or drain fluid.

The power calculation used to design the study was based on very low overall infection rates, assuming a reduction from 24% to 4% comparing placebo and study arms. The rate of infection found in the study was actually much higher than this, however with a large difference between the study groups, such that a statistically significant difference was still seen.

The study recorded a significant reduction in overall infection rate at 30-days with the Prepro symbiotic compared to placebo (22% versus 44%), *Klebsiella pneumoniae* being the most common organism. This seems to be particularly the case in blood-stream infections, but the data are not completely clear as some patients may have had more than one infection. There was no other significant difference in major complications seen, although the study had not been powered to detect small differences.

Despite the large reduction in infection rates, the study did not find that the use of probiotics reduced antibiotic use due to the low threshold for starting empirical treatment.

Previous, good quality, trials in liver transplantation have shown the benefit of probiotic and symbiotic preparations. This study adds significant supporting data to this.

